# Energy dissipation of nanoconfined hydration layer: Long-range hydration on the hydrophilic solid surface

**DOI:** 10.1038/srep06499

**Published:** 2014-09-30

**Authors:** Bongsu Kim, Soyoung Kwon, Hyosik Mun, Sangmin An, Wonho Jhe

**Affiliations:** 1 Department of Physics and Astronomy, Institute of Applied Physics, Seoul National University, Seoul 151-747, Korea; 2 Current address: National Institute of Standards and Technology, Gaithersburg, MD 20899, USA.

## Abstract

The hydration water layer (HWL), a ubiquitous form of water on the hydrophilic surfaces, exhibits anomalous characteristics different from bulk water and plays an important role in interfacial interactions. Despite extensive studies on the mechanical properties of HWL, one still lacks holistic understanding of its energy dissipation, which is critical to characterization of viscoelastic materials as well as identification of nanoscale dissipation processes. Here we address energy dissipation of nanoconfined HWL between two atomically flat hydrophilic solid surfaces (area of ~120 nm^2^) by small amplitude-modulation, noncontact atomic force microscopy. Based on the viscoelastic hydration-force model, the average dissipation energy is ~1 eV at the tapping amplitude (~0.1 nm) of the tip. In particular, we determine the accurate HWL thickness of ~6 layers of water molecules, as similarly observed on biological surfaces. Such a long-range interaction of HWL should be considered in the nanoscale phenomena such as friction, collision and self-assembly.

The hydration water layer (HWL) is a universal thin film of water formed on the hydrophilic surfaces in ambient conditions or in aqueous solutions^1^, whose characteristics is critical to better understanding of numerous HWL-related phenomena such as interfacial adhesion^2^, friction between surfaces^3^,^4^, filtration^5^, molecular transport in nanostructures^6^,^7^, molecular assembly of particles in liquid water^8^,^9^, and even biological inter-cellular processes^1^. The common responsible mechanism may be associated with the unique nanoscale characteristics of HWL in contrast to bulk water, which have been measured by various methods. For example, the ordered water structure at the surface-liquid interface was observed by infrared spectroscopy^10^ or X-ray crystallography^11^. The inter- and intra-molecular dynamic motion have been analysed by terahertz spectroscopy and molecular dynamic simulation^12^,^13^,^14^. In particular, the mechanical anomalies of HWL confined in the nanometric gap and the relevant interfacial forces have been studied by surface force apparatus (SFA)^1^,^4^,^15^ and scanning force microscopy (SFM)^2^,^3^,^16^, including the largely enhanced viscosity^3^, nonlinear viscoelasticity^17^ and non-squeeze-out fluidity^4^ of HWL.

Dissipation of energy is an important and critical physical process required for full characterization of nanoscale mechanics and dynamics of HWL. In general, the properties of energy dissipation are extensively analysed by the relevant force hysteresis^2^,^18^,^19^,^20^,^21^,^22^,^23^,^24^. However, it has been challenging to construct either the exact hydration-force hysteresis model due to the intricate hydration structure consisting of time-varying hydrogen-bond networks^25^,^26^, or the approximate force-hysteresis model due to the lack of the viscoelastic hydration-force formula. Without the explicit form of the hydration force, the elasticity and viscosity of HWL usually measured by atomic force microscopy (AFM) provide rather limited information for the energy dissipation of HWL^3^,^16^,^17^. Nonetheless, since the ‘average’ form of the viscoelastic hydration force is available^16^, one can address the overall response of the hydration structure by its first-order description of the energy dissipation of HWL, which can be done by performing measurements over the wide surface area of the AFM tip during detection time longer than relaxation time.

In this article, we first derive the explicit expression of the dissipated energy for the nanoconfined HWL based on the qualitative form of the viscoelastic hydration force^16^. We then demonstrate the validity of the calculated energy-dissipation formulas by comparing to the corresponding AFM experiments. In particular, it is remarkable that the quantitative energy dissipation analysis allows one to determine the exact thickness of HWL, beyond the well established information on the elastic and viscous properties of HWL. Experiments under various relative humidity (RH) show that HWL consists of about 6 layers of water molecules on the hydrophilic solid surface. This is thicker than the results of both computer simulation^27^,^28^ and experiments performed by the AFM-based sharp tip^25^,^26^,^29^, where the results indicate the hydration layer effect on the solid surface disappears at about 3 layers.

## Results

**Figure 1:**
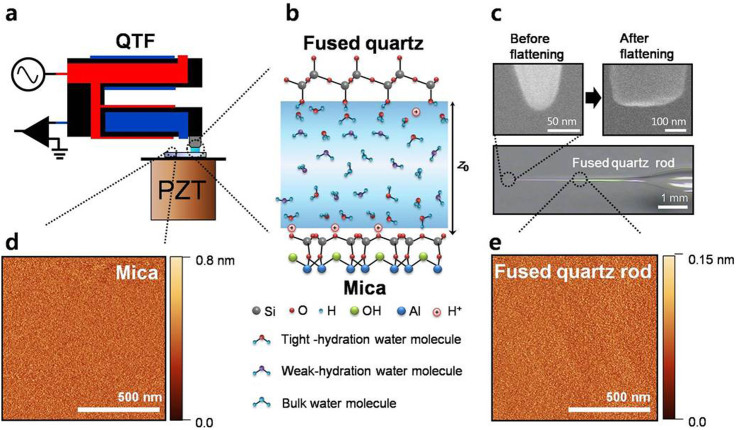
Experimental schematic and confined hydration water layer. (a) Experiments are performed using quartz tuning-fork (QTF) based AM-AFM. Mechanical properties of the confined hydration water layer (HWL) formed between the flattened fused quartz tip and mica surface are measured. (b) The water molecules are strongly hydrated close to the hydrophilic solid surfaces such as mica and quartz. Away from the surface, the molecules become increasingly weakly bound to the surface. (c) The fused quartz rod is fabricated by a mechanical laser puller (bottom) and the resulting sharp and round fused quartz tip is obtained (SEM image, top and left). The flattened tip used for experiments is obtained by repetitive gentle contacts to the mica surface (SEM image, top and right). (d) The root mean squared (RMS) surface roughness of mica is 0.39 Å measured by a commercial AFM. (e) The RMS surface roughness of the fused quartz rod is 0.14 Å. Both the flattened tip and mica are shown to be atomically flat.

**Figure 2:**
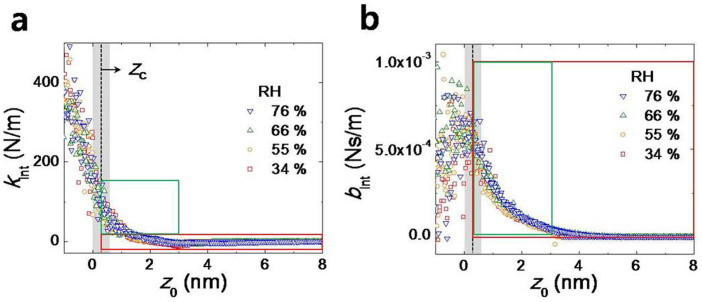
Experimental results and determination of contact point. (a) The measured elasticity and (b) damping coefficient resulting from the nanoconfined water (including the hydration water layer) between two atomically flat solid surfaces at various relative humidity (RH). The contact point (*z*_c_) is determined as the position where the damping coefficient shows abrupt changes as the tip approaches the substrate: *z*_c_ = 0.3 ± 0.3 nm that corresponds to the single monolayer thickness of the hydration layer. The data in the red and green box are presented in detail in Fig. 3.

By using the quartz tuning-fork (QTF) based, small amplitude-modulation (AM) AFM in the tapping-mode operation (Figs. 1a, 1b), we have measured the mechanical properties of HWL confined between the flattened fused quartz tip (top right in Fig. 1c) and the mica substrate. The root mean squared (RMS) surface roughness of mica (Fig. 1d) and fused quartz rod (Fig. 1e) has been measured as 0.39 Å and 0.14 Å, respectively, by a commercial AFM, which indicates that both surfaces are atomically flat (detailed information in Methods). Figure 2 presents the experimental effective elasticity (*k*_int_) and damping coefficient (*b*_int_) versus the distance *z*_0_ between the tip and substrate. The contact point (*z*_c_) is determined as the position where the standard deviation of the damping coefficient over repeated measurements abruptly increases by more than 5 times compared to that of the noncontact state as the tip approaches the substrate; *z*_c_ = 0.3 ± 0.3 nm which implies the fact that the monolayer of water molecules is not easily squeeze out in between the two flat hydrophilic surfaces.

**Figure 3:**
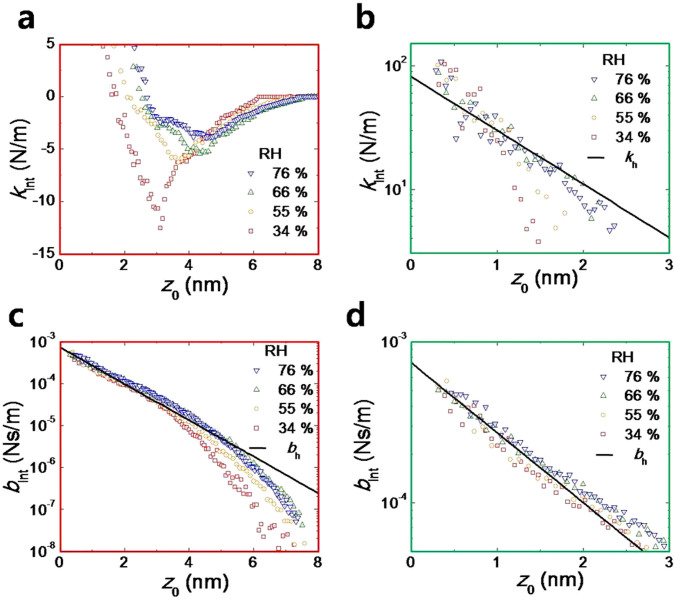
Variation of mechanical properties of the nanoconfined water. The data presented in the red (green) frame corresponds to the same data in the respective red (green) box in Fig. 2. (a), (b) Elasticity (*k*_int_) originates from the nanoconfined water that consists of both HWL and capillary nanomeniscus. (a) The humidity dependence appears where the distance *z*_0_ is larger than 1.2 nm, beyond which both the positive pressure due to the hydration layer and the negative Laplace pressure simultaneously contribute to *k*_int_. *k*_int_ becomes negative when the attractive Laplace pressure dominates at sufficiently large *z*_0_. (d) Below 1.2 nm of *z*_0_, *k*_int_ is positive and is attributed to the strongly repulsive hydration force due to the tightly bound HWL, which is independent of RH. (c), (d) Damping coefficient (*b*_int_) associated with both HWL and capillary nanomeniscus. (c) The humidity dependence appears where *z*_0_ is larger than 3.0 nm. Unlike the case of elasticity, it is ambiguous to determine where the dissipative effect of the hydration layer dominates or where the capillary force-hysteresis effect contributes. (d) *b*_int_ decreases with the same decay length as that of *k*_int_, in good agreement to equation (1), indicating the effects of HWL.

Figures 3a, b plot the effective elasticity *k*_int_ of Fig. 2a in detail (Fig. 3a) and in semi-logarithmic scale (Fig. 3b) versus several values of RH. Notice that the measured *k*_int_ combines the contributions of the nanoscale HWL as well as the capillary water-meniscus formed between the flattened fused quartz tip and the mica substrate. Figures 3c, 3d show the effective damping coefficient *b*_int_ of Fig. 2b in the semi-logarithmic scale. Interestingly, the humidity dependence of *k*_int_ disappears when *z*_0_ is less than ~1.2 ± 0.1 nm as shown in Fig. 3b, whereas the humidity dependence of *b*_int_ appears above ~3.3 ± 0.5 nm in Fig. 3c. The error values here are obtained by the standard deviation of the data obtained by repeated measurements.

The black lines in Fig. 3 represent the theoretical values of *k*_h_ and *b*_h_ that include only the contributions of HWL while excluding the effects of the capillary meniscus, given by^16^, 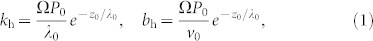
where Ω is the interaction area (π*r*_t_^2^) that is estimated as 120 nm^2^ from Fig. 1b (*r*_t_ is the radius of the interaction area of the flattened tip). *v*_0_, *P*_0_ and *λ*_0_ are the constant parameters associated with the velocity, pressure and characteristic length, obtained by curve fitting as 0.11 ± 0.02 mm/s, 1.78 ± 0.08 MPa and 1.01 ± 0.06 nm, respectively. Notice that *k*_h_ and *b*_h_ that result from HWL exhibit the decrease behaviour with the same characteristic length of the exponential decay as shown in Figs. 3b, 3d, which agree well to equation (1)^16^.

**Figure 4:**
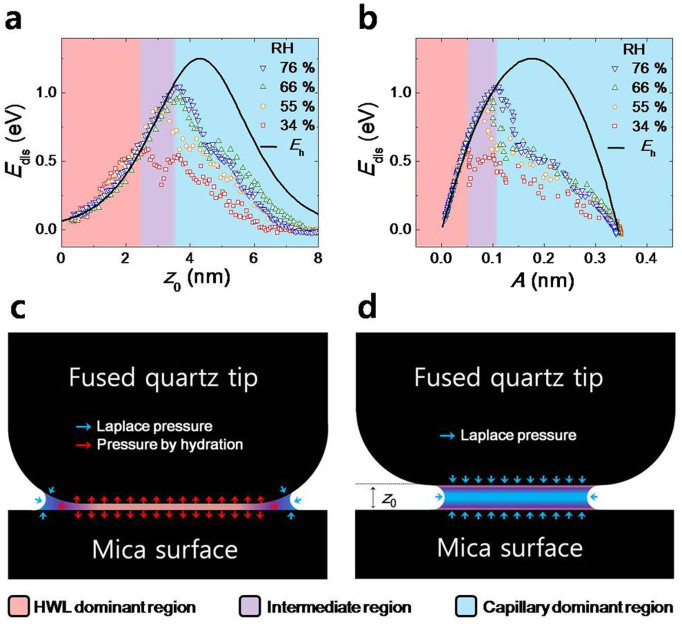
Energy dissipation of HWL and capillary force. (a) Energy dissipation (*E*_dis_) versus *z*_0_. The black curve represents the theoretical values (equation (2)) obtained from the viscoelastic hydration-force model, which shows good agreement to the experimental results in the hydration region (including both the tightly and weakly bound HWL). The repulsive hydration layer is maintained up until about 3.5 nm of *z*_0_ (that is, about 12 layer thickness of water molecules) at high RH. At low RH, the long-range hydration layer (or the weakly bound HWL) is collapsed by the attractive Laplace pressure effects, and thus only the tightly bound HWL remains. (b) *E*_dis_ versus oscillation amplitude of tip. The black curve shows the theoretical results and is well fitted with the experimental results in the hydration region. Notice that the bell shape of *E*_dis_ for the hydration layer exhibits the general behaviour similarly observed in other viscoelastic materials. (c) When the thickness of the nanoconfined water (including both HWL and capillary nanomeniscus) is sufficiently thin (under ~2.5 nm of *z*_0_), the tightly bound HWL formed on each surface exert positive (repulsive) pressure, as indicated by red arrows. Although the capillary meniscus may also exist at the rim of the tip, and provide negative (attractive) pressure (i.e., the Laplace pressure), its effect is dominated by the tightly bound HWL. (d) Above 3.5 nm of *z*_0_, the Laplace pressure effect dominates with insignificant effects of HWL observed. The red, purple and blue colour in Fig. 4 represent the region of the tightly hydrated layer, weakly hydrated layer, and capillary nanomeniscus, respectively.

Figures 4a, 4b present the dissipated energy per tapping cycle (*E*_dis_), versus the distance *z*_0_ (Fig. 4a) as well as versus the oscillation amplitude *A* of the tip (Fig. 4b). The black curves in Figs. 4a, 4b represent *E*_dis_ associated with HWL, which is derived as follows, 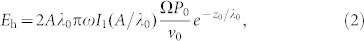
where *I*_1_(*x*) is the modified Bessel function of the first kind. Notice that in Fig. 4a, the experimental values of *E*_dis_ deviate from equation (2) above *z*_0_ in between 2.5 nm and 3.5 nm, depending on RH, which evidently indicates that the ‘repulsive’ effects of HWL disappear at the RH-dependent distance where the energy dissipation by the ‘attractive’ capillary force becomes dominant.

## Discussion

When the two HWLs, one on the flattened tip and the other on the mica substrate, become close and contact each other over the wide surface area, they exert the ‘average’ repulsive force, that is, the positive pressure on both surfaces as shown by the positive values of *k*_int_ both in equation (1) and in Figs. 3a, 3b^1^,^4^,^15^,^16^,^30^. In particular, under 1.2 nm of *z*_0_ (that is, within ~4 layers of water molecules), *k*_int_ is positive (Fig. 3b) resulting from the repulsive pressure in good agreement to equation (1), and is independent of RH (see also Fig. 4c). When *z*_0_ is larger than ~1.2 nm, on the other hand, *k*_int_ shows the RH-dependent deviation from equation (1) and becomes negative due to the attractive (capillary) force originating from the pressure difference, so called the Laplace pressure, between the interfacial liquid water and the surrounding vapour as shown in Fig. 3a (refer also to Fig. 4d)^30^,^31^,^32^. The Laplace pressure is the liquid-vapour surface tension divided by the radius of curvature of the water meniscus; Δ*P* = *γ*/*r*_k_, where *γ* and *r*_k_ are the surface tension and radius of curvature, respectively^30^,^32^. *r*_k_ corresponds to the thermodynamic Kelvin radius, which is closely connected to RH by the relation, 1/*r*_k_ ∝ ln(RH/100)^30^,^32^. Therefore, |*k*_int_| becomes larger (i.e., Δ*P* is larger) at the lower RH as shown in Fig. 3a (note that, however, |*k*_int_| increases with the increase of RH at *z*_0_ beyond 4 nm, which is attributed to the smaller size of the nanoconfined water bridge at the lower RH). Although the Laplace pressure effects appear above 1.2 nm distance at the low RH, the small HWL effects still exist until being completely dominated by the capillary-force effects.

In Figs. 3c, 3d, *b*_int_ also agrees with equation (1) at *z*_0_ less than 3 nm (that is, within ~10 layers of water molecules), and the RH dependence appears above 3 nm distance. Notice that the capillary force associated with the Laplace pressure should also affect *b*_int_ above 1.2 nm, as is the case for *k*_int_ (Figs. 3a, 3b)^20^. This additional effect on *b*_int_ may be probable because the tip that undergoes small-amplitude oscillatory motion generally results in the appropriate force hysteresis in the dissipative system^20^. As shown in Fig. 3c, such an RH-dependent effect appears at *z*_0_ above 3 nm, where *b*_int_ evidently deviates from equation (1). However, compared to the behaviours of *k*_int_, we find the results of *b*_int_ provide somewhat inconsistent information on the HWL characteristics as well as on the specific transition point from the HWL-dominating region to the capillary-dominating region. Therefore, this naturally leads one to investigate the energy dissipation process, in addition to *k*_int_ and *b*_int_, to have a full understanding of the dissipative property of HWL.

The dissipation energy (*E*_dis_) presents how far the HWL effects reach above the surface, which cannot be clearly determined in Fig. 3, as already discussed. Figure 4 shows the plot of *E*_dis_ versus *z*_0_ and *A* for several RH's. In Fig. 4a, *E*_dis_ is in excellent agreement to the theoretical values of equation (2) (black curve) under ~2.5 nm of *z*_0_ and is independent of RH, which corresponds to the HWL-dominating region that consists of the tightly bound HWL. However, beyond ~3.5 nm distance, *E*_dis_ deviates completely from the curve for all values of RH (almost saturating already at 76%), which consists of the bulk water region where the capillary force effect dominates with no explicit effects of HWL (refer to previous discussions on *k*_int_ and *b*_int_). Notice that the black curve represents *E*_h_ or equation (2) that is derived from the viscoelastic hydration-force model where only the effects of HWL alone are taken into account. At the higher RH, the distance where deviation occurs becomes farther, so that the ‘effective’ thickness of HWL is accordingly increased. However, it does not mean that all the water molecules in HWL are tightly bound at the high RH because HWL is formed only by the interaction between hydrophilic surface and water molecules, independently of the humidity. We attribute this intermediate region between 2.5 and 3.5 nm as resulting from ‘collapse’ of the effects of the weakly bound HWL due to the increasing Laplace pressure effect. In other words, in the intermediate region, some water molecules are maintained as weakly bound HWL while other water molecules behave similar to the bulk water. Thus, regardless of RH, the measured *E*_dis_ is smaller than the theoretical value *E*_h_ that is obtained on the assumption that all the water molecules form the HWL. As the effect of the weakly bound HWL disappears, *E*_dis_ deviates from equation (2) and thus becomes dependent on RH where *E*_dis_ originates from the capillary force hysteresis as discussed in *b*_int_.

The intriguing result is that the HWL exists thicker than usually expected. For example, at RH of 66% and 76%, the HWL is maintained until ~3.5 nm of *z*_0_ that corresponds to about 12 layer thickness of water molecules. In other words, HWL is consisted of ~6 layer thickness (~1.75 nm) of water molecules on each surface (that is, mica substrate and fused quartz tip), which includes the tightly as well as weakly bound HWL. The long range (~2.0 nm) hydration effect that appears on the stiff and atomically flat solid surface has not been addressed to date, although similar effect has been recently measured on the biological surfaces by terahertz spectroscopy^12^,^13^,^14^. For example, computer simulations of HWL on the solid surface have suggested the water molecules above the third molecular layer already behave similarly to the bulk water^27^,^28^, and experiments that measured the structure of hydration layer on the mica surface using the AFM-based sharp tip have also indicated that the hydration effect disappears even under 1 nm^25^,^26^,^29^. On the other hand, the interfacial force experiments using SFA often measured that the force between two flat surfaces violates the DLVO (Derjaguin and Landau, Verwey and Overbeek) theory beyond 2 nm distance^1^,^4^,^30^. Here, our results unambiguously show that the long-range HWL is formed on the stiff and atomically flat solid surfaces like the biological samples. Figure 4b presents *E*_dis_ versus amplitude. Up until the collapse of the hydration effect, the theoretical model value (black curve) agrees well to the experimental data, and *E*_dis_ versus amplitude shows the similar behaviours to other viscoelastic materials^18^,^22^. After the collapse, *E*_dis_ results from the capillary force hysteresis^20^ and decreases along with the amplitude increase in contrast to the hydration effect.

In summary, we have derived the dissipation energy formulas associated with HWL from the simple viscoelastic hydration-force model, and provided its quantitative experimental study using the noncontact AM-AFM. The energy dissipation analysis helps determine the accurate range where HWL plays a dominant role, so that the thickness of HWL can be unambiguously measured. At high RH, the effect of HWL is maintained up to 3.5 nm thickness, which indicates the long-range hydration layer of about 6 layers of water molecules is formed on the solid surface. At low RH, on the other hand, the Laplace pressure becomes strong and thus the long-range hydration layer collapses and only the tightly bound HWL remains below ~2.5 nm distance, independently of RH. Our results may provide new and significant insights for deeper understanding of the related phenomena, such as the nanoparticle self-assembly in ambient conditions. For example, the particle self-assembly process in air has been usually interpreted by the capillary interaction^33^,^34^,^35^,^36^ while the self-assembly of bio-molecule^8^ or nano-graphene^9^ in water has been understood dominantly by the HWL. However, when the particle size becomes nanometric scale, the interaction via the long-range HWL could be significant and thus should not be ignored. Moreover, it is also expected that the existence of the interplay between the long-range HWL and the Laplace pressure may contribute critically to collision as well as friction in the nanoscale systems such as the nanoelectromechanical system (NEMS) and colloidal nanoparticle system.

## Methods

### QTF-based AM-AFM

We measured the HWL properties between the fused quartz tip and mica using the quartz tuning-fork (QTF) based^37^,^38^ AM-AFM in the tapping-mode operation^39^,^40^. The fused quartz tip is strongly epoxied to one prong of the QTF, which allows noncontact and sensitive force-gradient (~0.01 N/m; this is 10 times smaller than the measured smallest elasticity of nanoconfined water) measurement at variable height due to its high stiffness (~20,000 N/m) and quality factor (~10,000) at the resonant frequency (~32 kHz) in ambient conditions. Figure 1a is the schematic diagram of the QTF that has two separate terminals, where each terminal is connected to the electrodes on the QTF. The measured amplitude and phase signals of the tip are converted to the effective elasticity (*k*_int_), damping coefficient (*b*_int_) and energy dissipation per cycle (*E*_dis_) of the HWL between the two surfaces^38^,^40^. The equation of motion and the solution of the tip motion are given as follows, 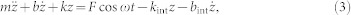

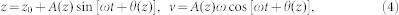
where *F* is the amplitude of the driving force, *m*, *b*, *k* and *ω* are the effective mass, damping coefficient, spring constant and driving angular frequency of QTF. And *z*_0_, *A* and *θ* are the mean separation between tip and substrate, oscillation amplitude and phase shift, respectively. Then *k*_int_, *b*_int_ and *E*_dis_ are given by^40^, 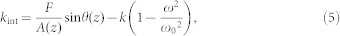

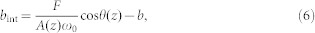

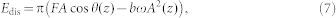
where *ω*_0_ is the resonance angular frequency of QTF. Notice that the trigonometric functions in equations (5), (6) and (7) should be adequately modified if different trigonometric functions are used in equations (3) and (4).

The flattened fused quartz tip that is attached to the QTF oscillates vertically at a given *z*_0_ while *z*_0_ itself is controlled by the piezoelectric transducer (PZT) on which the mica substrate is tightly fixed using an adhesive glue (Fig. 1a). Each data point shown in Figs. 2, 3, and 4 represent the time-average (for 300 ms) values obtained using the lock-in amplifier. The prepared fused quartz tip and mica substrate are placed in an air-tight chamber which is filled with both dry and wet nitrogen gas. The relative humidity in the chamber is controlled by the mixture ratio between the two gases.

### Fabrication of the flattened tip

The fabrication processes of the flattened fused quartz tip are as follows: (i) The sharp and round fused quartz tip (top and left in Fig. 1c) is fabricated by a laser-based mechanical puller (P-2000, Sutter Instruments Co.). We have measured the surface roughness of mica (Fig. 1d) and the fused quartz rod (Fig. 1e) by using contact-mode commercial AFM (Multimode SPM, Veeco Co.) where the diameter of the used cantilever tip is under 2 nm (SSS-NCLR-10, Nanosensors Co.). The root mean squared (RMS) roughness of the mica and the fused quartz is 0.39 Å and 0.14 Å, respectively. (ii) The round fused quartz tip is glued to one prong of the QTF using a strong epoxy (Araldite Rapid, Huntsman Advanced Materials Co.). (iii) The round fused quartz tip attached to the QTF is then forced to undergo repetitive, gentle contact against the atomically flat mica surface several hundred times. During this process, the round fused quartz tip becomes flattened. Then, without changing the alignment of the experimental setup, we perform experiments at several positions on the mica. The SEM image (top right of Fig. 1c) of the flattened tip was obtained after performing experiments. Notice that although the SEM image does not provide the nanometric details of the surface roughness of the flattened tip used in our experiments, it is still expected to exhibit the same subnanometric surface roughness, as indicated by the AFM image on the side surface of the fused quartz (Fig. 1e).

### Theoretical model of energy dissipation

The energy dissipated by an oscillating system is defined by, 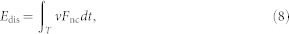
where *F*_nc_ is the dissipative force, *v* the velocity and *T* one period of the oscillator motion. The non-conservative dissipation force of the viscoelastic hydration model is given by^16^

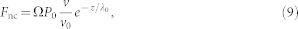
where Ω is the interaction area, *v*_0_, *P*_0_ and *λ*_0_ are the constants associated with velocity, pressure and characteristic length, respectively. Inserting equation (9) into equation (8), using equation (4) as the position and velocity of the oscillator, and then integrating over an oscillation period, one can derive the energy dissipation per period as given in equation (2). The constants (*v*_0_, *P*_0_ and *λ*_0_) in equation (2) can be obtained by fitting the experimental *k*_int_ and *b*_int_ data to the effective elasticity (*k*_h_) and damping coefficient (*b*_h_) for the viscoelastic hydration force model^16^. Since *k*_int_ and *b*_int_ in equations (5) and (6) are matched to equation (1) by fitting, the amplitude *A* can be obtained from equations (5) and (6) as, 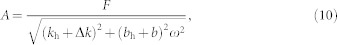
where 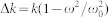 is the detuning (In supplementary information, the theoretical values (equation (10)) are compared to the experimental data.) As a result, one can obtain analytically the dissipation energy of HWL using the viscoelastic hydration force model.

## Author Contributions

B.K. and W.J. designed and directed the research. B.K., S.K. and W.J. wrote the manuscript. B.K. and S.K. prepared the samples and tips, and carried out the experiments. B.K. and W.J. developed the theoretical model of the energy dissipation process due to the hydration layer. H.M. measured the roughness of mica and fused quartz rod. S.A. helped experiment and provided invaluable discussions.

## Supplementary Material

Supplementary Information Supplementary
