# Supplementary material for: Energy dissipation of nanoconfined hydration layer: Long-range hydration on the hydrophilic solid surface
Source: Sci Rep. 2014 Sep 30;4:6499. doi: 10.1038/srep06499 (PMC4179125; doi:10.1038/srep06499)
Supplement: Supplementary Information — Supplementary [file srep06499-s1.pdf]

Energy dissipation of nanoconfined hydration layer: Long-range hydration on the hydrophilic solid surface

Supplementary information

Bongsu Kim, Soyoung Kwon, Hyosik Mun, Sangmin An†, Wonho Jhe

Department of Physics and Astronomy, Institute of Applied Physics, Seoul National University, Seoul 151-747, Korea

†*Present address: National Institute of Standards and Technology, MD 20899,USA*

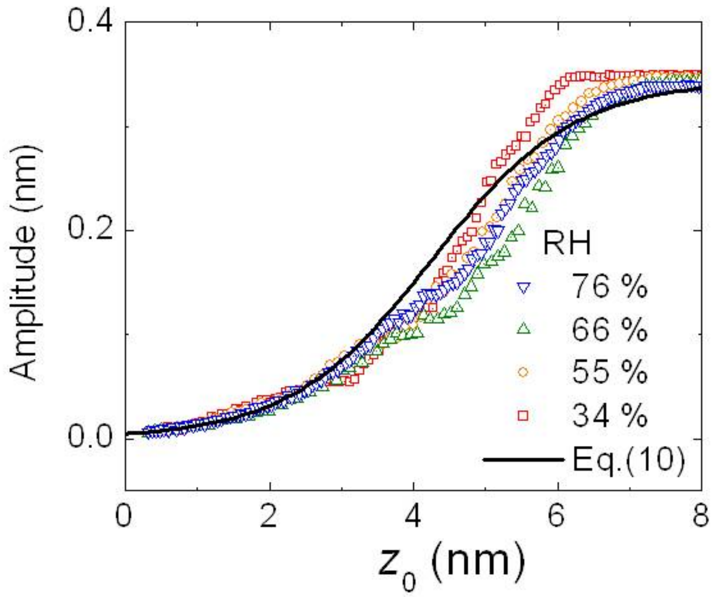

Figure S1. Oscillation amplitude *A* versus inter-surface distance z_0. The black curve represents the theoretical value based on the viscoelastic hydration-force model (equation (10)) and is in good agreement with experimental results in the hydration region (below ~ 3.5 nm).
